# The Effect on Treatment Adherence of Administering Drugs as Fixed-Dose Combinations versus as Separate Pills: Systematic Review and Meta-Analysis

**DOI:** 10.1155/2014/967073

**Published:** 2014-09-04

**Authors:** Katy A. van Galen, Jeannine F. Nellen, Pythia T. Nieuwkerk

**Affiliations:** ^1^Division of Infectious Diseases, Tropical Medicine and AIDS, Academic Medical Center, Amsterdam, The Netherlands; ^2^Department of Medical Psychology (J3-219-1), Academic Medical Center, Meibergdreef 9, 1105 AZ Amsterdam, The Netherlands

## Abstract

Administering drugs as fixed-dose combinations (FDCs) versus the same active drugs administered as separate pills is assumed to enhance treatment adherence. We synthesized evidence from randomized controlled trials (RCTs) about the effect of FDCs versus separate pills on adherence. We searched PubMed for RCTs comparing a FDC with the same active drugs administered as separate pills, including a quantitative estimate of treatment adherence, without restriction to medical condition. The odds ratio (OR) of optimal adherence with FDCs versus separate pills was used as common effect size and aggregated into a pooled effect estimate using a random effect model with inverse variance weights. Out of 1258 articles screened, only six studies fulfilled inclusion criteria. Across medical conditions, administering drugs as FDC significantly increased the likelihood of optimal adherence (OR 1.33 (95% CI, 1.03–1.71)). Within subgroups of specific medical conditions, the favourable effect of FDCs on adherence was of borderline statistical significance for HIV infection only (OR 1.46 (95% CI, 1.00–2.13)). We observed a remarkable paucity of RCTs comparing the effect on adherence of administering drugs as FDC versus as separate pills. Administering drugs as FDC improved medication adherence. However, this conclusion is based on a limited number of RCTs only.

## 1. Introduction

Adherence to combination antiretroviral therapy (cART) is a key predictor of antiretroviral treatment success and survival [1, 2]. Past research has identified treatment complexity as one of the factors contributing to low levels of adherence [3–6]. The complexity of a patient's medication regimen may refer to the number of prescribed medications per day, that is, pill burden, the daily dosage frequency, and special administration instructions [7]. The vast majority of studies investigating the effect of treatment simplification on adherence have focused on the effect of once daily dosing versus twice daily dosing, generally yielding better adherence rates for once daily regimens [8, 9]. Another simplification strategy consists of the use of fixed-dose combinations (FDCs). FDCs combine two or more active drugs in one single-tablet or capsule. The reduction in pill burden associated with the use of FDCs is assumed to improve patient adherence. Yet, randomized studies investigating this possible benefit of FDCs are scarce as FDCs are usually approved on the basis of safety and bioequivalence rather than noninferiority to the component regimens.

The question to what extent FDCs lead to better adherence than the same active drugs administered as separate pills has nowadays become highly relevant. The patents of several antiretroviral drugs have recently expired and others will expire in upcoming years. This offers the potential for the replacement of branded FDCs by cheaper generics containing the same active drugs administered as separate pills [10, 11]. It was recently shown that decoupling of branded FDCs into separate generic and branded drugs could result in considerable cost savings [12, 13]. Current pressures to control healthcare expenditures in many countries could encourage the decoupling of branded FDCs. Possibly, these cost savings come at the expense of decreased levels of adherence [14]. Knowledge about the quantitative effect on adherence of administering drugs as FDCs versus as separate pills of the same active drugs could help making this trade-off. The objective of the present study was to summarize and synthesize existing research evidence from randomized controlled trials about the effect on treatment adherence of administering drugs as FDCs versus the same active drugs administered as separate pills.

## 2. Methods

We searched the PubMed database from inception to December 2012 for papers fulfilling the following inclusion criteria: (1) randomized controlled trial, (2) comparing a FDC with the same active drugs administered as separate pills, (3) administration route being oral, (4) medications being self-administered, (5) endpoints of the study included a quantitative estimate of treatment adherence, and (6) the paper being published in English language. We included papers without restriction in type of diseases and/or medications.

We used the following search strategy: (Therapy/broad[filter]) and ((((Fixed-dose combination[tiab] or “Drug Combinations”[Majr] or drug combination^∗^[tiab] or single pill combination^∗^[tiab] or polypill[tiab])) and (“Patient Compliance”[Majr] or compliance^∗^[tiab] or adherence^∗^[tiab] or nonadherence^∗^[tiab] or nonadherence^∗^[tiab] or noncompliance^∗^[tiab] or noncompliance^∗^[tiab] or “Treatment Outcome”[Mesh] or treatment outcome^∗^[tiab]))).

If the title and the abstract met inclusion criteria, the full text of the article was retrieved and the article was included if all the inclusion criteria were met. We screened the reference lists of papers identified by our search strategy to find additional potentially eligible studies.

Two authors (KG and PN) independently extracted data from each study that fulfilled inclusion criteria. We extracted the following information: name of the first author, year of publication, sample size, country in which the study was conducted, medical condition for which treatment was prescribed, medicines administered in the intervention group (FDC) and in the control group (separate pills), duration of follow-up, and adherence assessment method.

We used the odds ratio (OR) of optimal adherence with FDCs versus separate pills as common effect size. We calculated the *I* ^2^ index as measure of between study heterogeneity in effect sizes. We used a random effect model with inverse variance weights to aggregate individual effect sizes into a pooled effect estimate with 95% confidence limits using Review Manager 5.2. We repeated this analysis for subgroups of different medical conditions. Additionally, we repeated the analysis with the study on hypertension being removed from the analysis. We examined the presence of possible publication bias by visual inspection of funnel plots and by investigating the statistical significance of Egger's regression intercept using Comprehensive Meta-Analysis version 2.

## 3. Results

**Figure 1:**
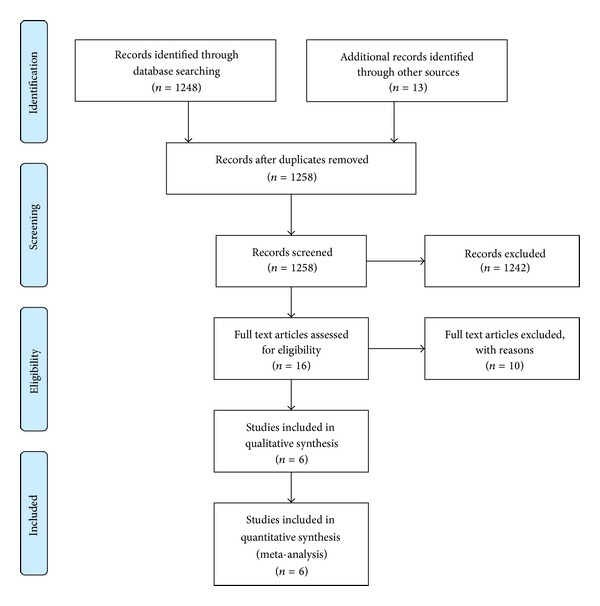
Flow diagram.

A total of 1258 potentially relevant articles were identified and were subsequently screened (Figure 1). A total of 16 full text articles were assessed for eligibility. Of these, ten articles were excluded for the following reasons: medicines were not self-administered (*n* = 6), separate pills had not the same active drugs as FDCs (*n* = 3), and the study design was not a randomized controlled trial (*n* = 1). The characteristics of the 6 included studies are shown in Table 1.

**Figure 2:**
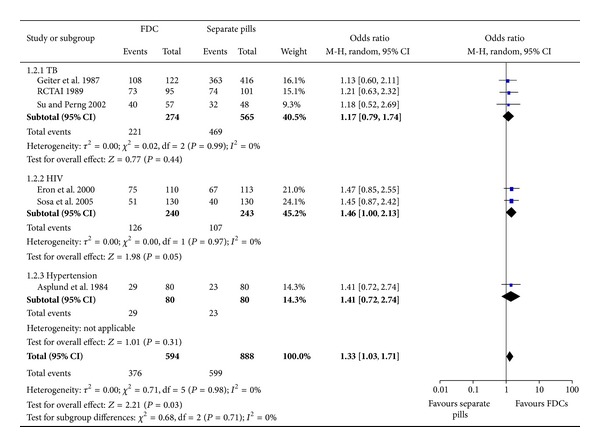
Effect of FDCs versus separate pills on treatment adherence.

Results of the quantitative pooling of effect sizes are shown in Figure 2. Across medical conditions, administering drugs as FDC versus as separate pills significantly increased the likelihood of optimal adherence. Within subgroups of specific medical conditions, the favorable effect of FDCs versus separate pills on adherence was statistically significant for HIV infection only, although results for TB and hypertension were in the same direction. After removing the study on hypertension, administering drugs as FDC still significantly increased the likelihood of optimal adherence (OR 1.31 (95% CI, 1.00–1.73)). Neither the funnel plot nor Eggers regression intercept (*P* = 0.16) was indicative of publication bias (data not shown).

## 4. Discussion

In our meta-analysis of randomized controlled trials comparing the effect on treatment adherence of administering drugs as FDCs versus as separate pills, FDCs resulted in improved adherence compared with separate pills. Nevertheless, the result was based on a limited number of studies only, as we observed a remarkable paucity of RCTs comparing the effect on adherence of administering drugs as FDC versus as separate pills. Across various medical conditions, this effect of FDCs on adherence was statistically significant for the treatment of HIV infection only, although the effect for other medical conditions was in the same direction.

Our results are in line with a previous meta-analysis investigating the effect of fixed dose combinations on medication compliance [21]. This previous meta-analysis found a substantial reduction in the risk of nonadherence for FDCs compared with non-FDCs. Results from the present study are in the same direction but are less pronounced. This previous meta-analysis included not only randomized controlled trials, but also retrospective observational studies. Nonrandomized studies have been shown to overestimate intervention effects [22]. When the previous meta-analysis was restricted to randomized studies, the favourable effect of FDCs diminished and was no longer statistically significant. Nonrandomized studies are more susceptible to unaccounted confounding than randomized studies. Combining evidence from randomized and nonrandomized studies studies is not recommended [23]. We therefore included only randomized trials in the present meta-analysis.

A number of observational studies in HIV infection have compared adherence and/or virological response with single versus multitablet regimens [24–28]. In one study, switching patients from multitablet regimens to single-tablet regimens resulted in improved adherence while maintaining virological response [24]. In another study, switching patients from single-tablet regimens to multitablet regimens did not result in a diminished virological response [25]. Some studies comparing adherence and/or virological response among patients on single versus multitablet regimens have found significantly higher adherence [26, 27] and/or a more favorable virological response in the single-tablet groups [26, 28] compared with the multitablet groups, whereas others have found no difference in adherence [29].

Studies investigating the association between daily pill burden and virological response have demonstrated a significant relationship between a higher pill burden and a lower virological suppression rate [30, 31] that was independent of daily dosing frequency [31]. Most of these observational studies suggest that single-tablet regimens result in higher levels of adherence and improved virological response. However, interpretation of the results of these studies is complicated by methodological limitations. The switch studies did not include a comparison group. All of the observational studies may have been susceptible to unmeasured confounding in particular channelling bias. Because the selection of treatments was not random in these studies and was most likely determined by patient and physician characteristics, the observed effects of single-tablet regimens in comparison with multitablet regimens may have been influenced by factors other than the treatment. For example, physician may be more likely to prescribe multitablet boosted protease inhibitor based regimens to patients with higher baseline viral load or those judged less likely to be adherent, as these drugs are generally more “forgiving” of missed doses. Another example is that patients with prior virological treatment failure may be more likely to be prescribed multitablet regimens and to have higher viral loads.

The present study adds to these previous studies that evidence from randomized controlled trials of FDCs versus separate pills also show improved levels of adherence for FDCs. The present study has several limitations. First, the number of randomized controlled trials comparing FDCs with separate pills is small. Therefore, we were able to include only a limited number of trials in our meta-analysis and we choose to include trials irrespective of the medical condition for which treatments were prescribed. Second, for one of the trials conducted in HIV infection it was impossible to distinguish the effect of FDCs from the effect of daily dosing frequency [16]. Third, we searched for papers in the PubMed database only and restricted our search to full text papers. Fourth, studies included in our meta-analysis have used a variety of adherence assessment methods. Nevertheless, the statistical heterogeneity between studies in effect sizes was low.

In conclusion, our meta-analysis of randomized controlled trials yielded an increased likelihood of improved adherence for FDCs compared with separate pills. However, this conclusion is based on a limited number of randomized controlled trials.

Our findings suggest that decoupling FDCs into separate pills could lead to lower levels of adherence. We, therefore, recommend careful monitoring of patients who are switched from FDCs to multitablet regimens for economic reasons.

## Figures and Tables

**Table 1:** Characteristics of included studies.

Study	*n*	Medical condition	Country	FDC arm	Separate pills arm	Duration of follow-up	Adherence assessment method
Eron et al., 2000 [15]	223	HIV infection	USA	FCD of lamivudine and zidovudine in combination with a PI	Separate lamivudine, zidovudine, and a PI	4 months	Self-reported adherence

Sosa et al., 2005 [16]	236	HIV infection	USA, Panama, Costa Rica, Puerto Rico	Once daily FDC of abacavir and lamivudine in combination with a PI or NNRTI	Twice daily separate abacavir and lamivudine in combination with a PI or NNRTI	12 months	Pill count

Geiter et al., 1987 [17]	538	Pulmonary tuberculosis	USA	FDC of isoniazid, rifampicin, and pyrazinamide for 2 months followed by FDC of isoniazid and rifampin for 4 months	Separate isoniazid, rifampicin, and pyrazinamide for 2 months followed by separate isoniazid and rifampin for 4 months	6 months	Self-reported adherence, pill count, and appointment keeping

Su and Perng, 2002 [18]	105	Pulmonary tuberculosis	Taiwan	FDC of isoniazid, rifampicin, pyrazinamide and ethambutol for 2 months, followed by FDC of isoniazid and rifampicin for 4 months	Separate isoniazid, rifampicin, pyrazinamide, and ethambutol for 2 months, followed by separate isoniazid and rifampicin for 4 months	12 months	Adherence with clinic visits

RCTAI, 1989 [19]	196	Pulmonary tuberculosis	India	FDC of isoniazid, rifampicin, and pyrazinamide for 8 weeks followed by FDC of isoniazid and rifampin for 18 weeks	Separate isoniazid, rifampicin, and pyrazinamide for 8 weeks followed by separate of isoniazid and rifampin for 18 weeks	26 weeks	Home based pill counts

Asplund et al., 1984 [20]	160	Hypertension	Sweden	FDC of pindolol and clopamide	Separate pindolol and clopamide	8 months	Pill count
